# Sinks and Sources of Intracellular Nitrate in Gromiids

**DOI:** 10.3389/fmicb.2017.00617

**Published:** 2017-04-20

**Authors:** Signe Høgslund, Tomas Cedhagen, Samuel S. Bowser, Nils Risgaard-Petersen

**Affiliations:** ^1^Department of Bioscience, Aarhus UniversityAarhus, Denmark; ^2^Wadsworth Center, New York State Department of Health, AlbanyNY, USA; ^3^Center for Geomicrobiology, Aarhus UniversityAarhus, Denmark

**Keywords:** Gromia, nitrate, denitrification, nitrification, aerobic respiration, protist, endobionts

## Abstract

A substantial nitrate pool is stored within living cells in various benthic marine environments. The fate of this bioavailable nitrogen differs according to the organisms managing the intracellular nitrate (ICN). While some light has been shed on the nitrate carried by diatoms and foraminiferans, no study has so far followed the nitrate kept by gromiids. Gromiids are large protists and their ICN concentration can exceed 1000x the ambient nitrate concentration. In the present study we investigated gromiids from diverse habitats and showed that they contained nitrate at concentrations ranging from 1 to 370 mM. We used ^15^N tracer techniques to investigate the source of this ICN, and found that it originated both from active nitrate uptake from the environment and from intracellular production, most likely through bacterial nitrification. Microsensor measurements showed that part of the ICN was denitrified to N_2_ when gromiids were exposed to anoxia. Denitrification seemed to be mediated by endobiotic bacteria because antibiotics inhibited denitrification activity. The active uptake of nitrate suggests that ICN plays a role in gromiid physiology and is not merely a consequence of the gromiid hosting a diverse bacterial community. Measurements of aerobic respiration rates and modeling of oxygen consumption by individual gromiid cells suggested that gromiids may occasionally turn anoxic by their own respiration activity and thus need strategies for coping with this self-inflicted anoxia.

## Introduction

Bioavailable nitrogen, in form of nitrate, is present in the upper millimeters of the sediment pore water where it acts as a key nutrient and electron acceptor in many benthic marine ecosystems. A substantial nitrate pool is, however, also maintained by eukaryotic microbes inhabiting the sediment environment. This intracellular nitrate (ICN) can comprise a nitrate pool that exceeds pore water pools by orders of magnitude (Mussmann et al., 2003; Høgslund et al., 2010; Kamp et al., 2015; Garcia-Robledo et al., 2016). ICN is found in diverse prokaryotic and eukaryotic microbes, including the large white sulfur bacteria (Fossing et al., 1995; McHatton et al., 1996), foraminifera (Risgaard-Petersen et al., 2006; Piña-Ochoa et al., 2010), and diatoms (Dortch et al., 1984; Lomstein et al., 1990; Kamp et al., 2011, 2013), which all store nitrate within their cells at concentrations of 10–100 mM. It has become increasingly clear that ICN is a highly dynamic nitrate pool, and a significant component of the nitrogen cycle (Kamp et al., 2015). Observations from a study site in the Wadden Sea, Germany demonstrate, for instance, that 20–40% of the nitrate entering sediments enter an ICN pool, and that about 30% of this ICN is respired by eukaryotes (Marchant et al., 2014). Further, estimates of denitrification of ICN in foraminifers indicate that up to 90% of the general loss of nitrogen as nitrogen gas from some sediment is based on respiration of ICN (Piña-Ochoa et al., 2010; Dale et al., 2016) and reviewed by Kamp et al. (2015).

The fate of ICN depends on the organism carrying the nitrate. Large sulfur bacteria like *Beggiatoa* and *Thioploca* (Otte et al., 1999; Sayama et al., 2005; Høgslund et al., 2009), as well as certain diatoms (Kamp et al., 2011, 2013), convert their ICN to ammonium through dissimilatory nitrate reduction to ammonium (DNRA). Nitrate stored by foraminifera is converted to either N_2_ or N_2_O through denitrification by the foraminifera (Risgaard-Petersen et al., 2006; Bernhard et al., 2012a) or through denitrification carried out by bacterial endosymbionts (Bernhard et al., 2012b).

**FIGURE 1:**
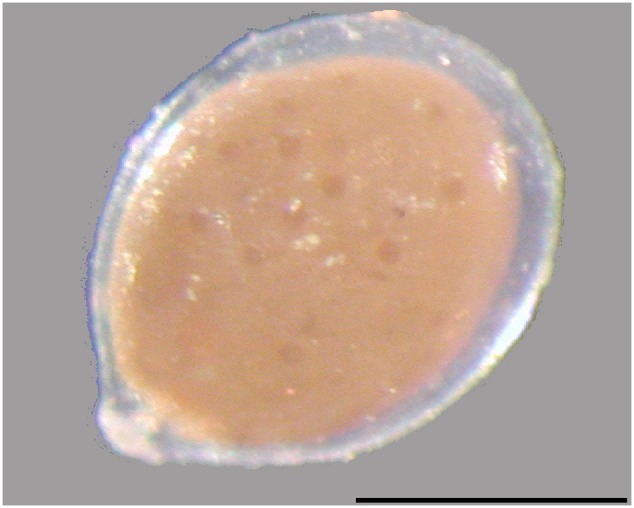
**Gromiid cells are surrounded by a proteinaceous test which encloses a cytoplasm filled with stercomata.** The oral capsule is seen at the lower left. Scale bar 500 μm.

The database on ICN-storing microorganisms is far from complete, and this limits our understanding of the significance and turnover of ICN in the environment. Specimens of the protistan genus *Gromia* carry ICN in millimolar concentrations (Piña-Ochoa et al., 2010), but the prevalence of the trait within this genus and fate of its ICN is unknown. Gromiids are protists, that are typically around or below one millimeter in diameter (Arnold, 1972) but specimens with diameters of 30 mm has been reported from several deep-sea sites (Gooday et al., 2000; Matz et al., 2008). Gromiids superficially resemble some allogromiid foraminifera, but *Gromia* is a well-defined genus within the Rhizaria and distinct from the foraminifera (Burki et al., 2010; Sierra et al., 2013). The organism is surrounded by a proteinaceous transparent test, which encapsulates a cell body filled with sediment and debris packaged as discrete structures termed stercomata (**Figure 1**). The cell extends granulofilose pseudopods, protruding through an aperture which is surrounded by an oral capsule; both of these features comprise characteristic features of the group (Hedley and Bertaud, 1962; Hedley and Wakefield, 1969). They occupy various habitats from intertidal mudflats, rocky bottoms in the arctic, sandy sediments off the Bahamas, bathyal and nearshore to abyssal seafloor in the Southern Ocean and sediments under oxygen minimum zones (Jepps, 1926; Hedley and Bertaud, 1962; Arnold, 1972; Gooday et al., 2005; Gooday and Bowser, 2005; Matz et al., 2008; da Silva and Gooday, 2009; Rothe et al., 2011).

In the present study we investigate whether nitrate storage is a general trend among gromiids by analyzing the nitrate content in individual specimens from different environments. We also study extracellular and intracellular sources of ICN in the gromiids using ^15^N techniques. Sinks for ICN are examined using microsensor measurements of denitrification rates, and the role of endobiont bacteria in the nitrate turnover is addressed by antibiotic amendments. The storage and use of ICN is discussed in relation to the life mode of gromiids, respiration activity and intracellular oxygenation status measured by oxygen microelectrodes. By elucidating nitrogen dynamics associated with gromiids we aim to contribute to the understanding of the impact of ICN on marine nitrogen cycles, and to the ecology of nitrate-storing eukaryotes.

## Materials and Methods

### Gromiid Sampling

Gromiids were sampled at four different locations: Uummannaq, Greenland 70° 33′48.93″N, 51° 07′ 04.38″V Skagerak, Denmark 57° 47′05.48″N, 9° 45′55.47″ Ø Kattegat, Denmark 56°26′36.05″N, 10°57′32.80″Ø, Lillebælt, Denmark 55° 24′ 24.11″N, 9° 39′35.83″Ø and eastern McMurdo Sound, Antarctica 77° 51′06.90″ S, 166° 39′83.10″ E. Antarctic gromiids were collected from sediments sampled by SCUBA divers at 2–3.5 m water depth near the McMurdo Station jetty. Gromiids sampled from Danish and Greenlandic locations were retrieved from hapteras of *Laminaria* collected at 2–5 m water depths. The hapter were scrubbed in a bucket of seawater and the resulting suspension was passed through a series of sieves (mesh size: 63 μm, 500 μm, 1 mm and 2 mm) as described by Arnold (1972). The content of each sieve fraction was transferred to clean buckets and overlain with 2 mm of air saturated seawater. Samples were stored at 6°C until further processing.

### Intracellular Nitrate

Intracellular nitrate content was measured on individuals sampled at Ummannaq, Greenland (*n* = 10), Kattegat, Denmark (*n* = 18), Skagerrak, Denmark (*n* = 3), and McMurdo Sound, Antarctica (*n* = 21). Individual gromiids were picked within 4–24 h after sampling, then cleaned and measured (length and width) using a dissection microscope. Single gromiids were then transferred to 0.5 ml Eppendorf tube and frozen to promote cell lysis. Prior to analysis, the gromiids were thawed directly in the tube; ground using a small glass spatula, then 100 μl milli-Q water was added to the tube to extract the nitrate and nitrite from the ground gromiid. Nitrate and nitrite were measured as NO (Braman and Hendrix, 1989) with a chemiluminescence detector (Model CLD 86; Eco Physics AG) on 25–50 μl subsamples of the extract as described in Høgslund et al. (2008). Intracellular concentrations were calculated from the total nitrate content and the biovolume of the individual gromiid. The biovolume was determined from the cellular dimensions using the best representative geometric shape, which was either a sphere or a cylinder.

Shapiro Will test (shapiro.test function; R Development Core Team, 2014) was used to test if the intracellular concentration and content of nitrate within the populations from each of the sampled regions followed a normal or a log-normal distribution. It was found that the ICN content and concentration was log-normal distributed within the populations. Therefore ICN content and concentration are reported as the geometric mean for each of the geographic regions sampled with upper and lower 95% confidence limits given (Limpert et al., 2001; Limpert and Stahel, 2011).

### Uptake of Extracellular Nitrate

The ability to take up nitrate from the environment was tested in ^15^NO_3_^-^ experiments on 22 single gromiids sampled from Kattegat, Denmark. Material retrieved from haptera was kept for 5 days at 6°C until sorting and cleaning. The gromiids were cleaned in 30‰ artificial nitrate-free sea water (Red Sea Coral pro salt; Red Sea Fish Pham LTD, Israel), then pre-incubated at 10°C for 1 week in a 6 ml glass vial (Exetainer Labco, UK) with N_2_ – CO_2_ (400 ppm CO_2_) purged nitrate-free artificial seawater to exhaust the ICN pool. The gromiids were then transferred to a Petri dish and covered with two mm of air-saturated artificial seawater (12 ml) with ^15^N-labled nitrate (atom % 99) added to a final concentration of 120 μM. The Petri dish was kept at 10°C with a lid to prevent evaporation. A subset of specimens was picked for ^15^NO_3_^-^ determinations at the start of the pre-incubation period (*n* = 4); another right after transfer to the Petri dish with ^15^NO_3_^-^ supplemented water (*n* = 4); and others after one (*n* = 6) and 2 weeks (*n* = 8) incubation in the ^15^NO_3_^-^ water. Each picked individual was rinsed three times in nitrate-free artificial seawater to assure that no ^15^NO_3_^-^ from the incubation media was transferred with the gromiid, then its cellular dimensions were measured, whereafter it was transferred to a 0.5 ml Eppendorf tube and frozen at -20°C to promote cell lysis. Nitrate from each gromiid was extracted with 100 μl miliQ water as described above. The concentration of ^14^NO_3_^-^ and ^15^NO_3_^-^ in the extract was determined by combined ion chromatography-mass spectrometry on a Dionex ion Chromatograph 3000 system coupled to Thermo Surveyor Quadrupole Mass Spectrometer equipped with Electro Spray ionization. A trap column, Dionex UTAC-ULP1, was used to concentrate the sample prior to injection into the mass spectrometer. Pearson product-moment correlation analysis [R-function cor.test(); R Development Core Team, 2014] was used to test for significant correlations between incubation time and ^15^N-abundance in the ICN pool of the gromiids; this analysis was performed on log transformed ^15^N data to meet the requirement of normal distribution.

### Intracellular Production of Intracellular Nitrate

The capacity for intracellular production of ICN via nitrification was tested in ^15^N- and acetylene inhibition experiments on gromiids collected in the Kattegat. Material retrieved from hapera was kept for 2 days at 6°C until sorting and cleaning for individual gromiids. Initially, 118 specimens were pre-incubated anaerobically in an Exetainer for 3 days as described above. Thereafter, 52 individuals were distributed into six 6 ml Exetainers containing 250 μl nitrate free artificial seawater with 5 μM ^15^NH_4_^+^. Another 68 individuals were distributed into six 6 ml Exetainers containing 250 μl nitrate free artificial seawater without ammonium. After capping, half of the Exetainers from both treatments were supplemented with 10 Pa acetylene, which at low concentrations (0.1–10 Pa) irreversibly inhibits ammonium mono-oxidase (Berg et al., 1982). Every 2nd day during the 2 week incubation period each Exetainer was uncapped, gently shaken to ensure aerobic conditions, and acetylene concentrations were restored by supplementing with 10 Pa acetylene before resealing. Individual gromiids were picked for nitrate analysis right after transfer to the oxic media in the Exetainers and after 1 and 2 weeks of incubation. After picking the gromiids, they were rinsed in nitrate-free artificial water, their cell dimensions measured and their content of intracellular ^15^NO_3_^-^ and ^14^NO_3_^-^ were determined as described above. Nitrate concentration in the incubation media was always below 2 μM and the ^15^N-labeling below 1%. Pearson product-moment correlation analysis [R-function cor.test(); R Development Core Team, 2014] was used to test relations between incubation time and the magnitude and ^15^N-labeling of the ICN pools. Student’s *t*-test [R-function t.test(); R Development Core Team, 2014] was used to test for differences in abundance and ^15^N labeling of the ICN pool among gromiids incubated in the presence or absence of acetylene. All analyses were performed on log transformed ICN data to meet the requirement of normal distribution.

### Aerobic Respiration Rates and Denitrification Activity

Oxygen consumption and denitrification rates were measured on gromiids collected from Uummannaq, Greenland and the Lillebælt, Denmark. The specimens used were sampled from source material kept at 6°C in the laboratory for 5 days (Lillebælt samples) or 1 month (Greenlandic samples) with air saturated nitrate supplemented (100 μM) artificial sea water. In our experience, viable gromiids can be kept for at least 6 months without loss of ICN or turgor pressure at these conditions.

**FIGURE 2:**
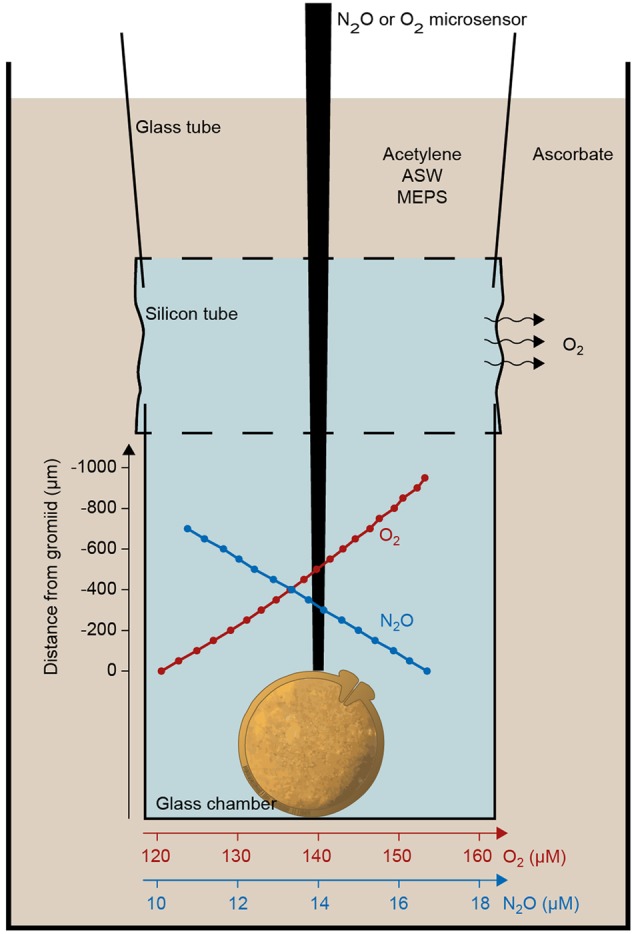
**Experimental setup for microsensor measurements of oxygen and nitrous oxide profiles above single gromiid cells.** An example of oxygen and a nitrous oxide profile is shown in red and blue. Oxygen profiles were measured during oxic conditions where ascorbate was replaced by artificial seawater.

Measurements were carried out in glass microtubes (**Figure 2**), each containing 1–6 specimens in artificial nitrate-free, HEPES-buffered artificial seawater (Høgslund et al., 2008; Piña-Ochoa et al., 2010; Geslin et al., 2011). The microtubes were mounted at one end of a silicon tube. A glass tube was inserted in the other end of the silicone tube, and this glass tube was affixed to a cuvette (**Figure 2**). The silicone tube allowed free diffusion of oxygen into the incubation media and oxygen concentration in the chambers could be controlled by adding either oxic seawater (oxic incubations) or alkaline ascorbate to the cuvette (anoxic incubations).

Oxygen consumption rates were estimated from the concentration gradient of oxygen in oxic chambers measured with an O_2_ microsensor (Revsbech, 1989) and determined as the product of the surface area of the glass microtube and the O_2_ flux, the latter determined from the measured O_2_ concentration profiles by means of Fick’s first law of diffusion. The salinity (35 ‰)- and temperature (7°C)- corrected diffusion coefficient used in these calculations was retrieved with the function diffcoeff() from the R-package “marelac” (Soetaert et al., 2016).

Denitrification measurements were based on the acetylene inhibition method (Smith et al., 1978). Acetylene blocks the last step in the denitrification pathway, the reduction of N_2_O to N_2_ and denitrification of ICN may therefore be quantified by measurement of N_2_O production. N_2_O production rates were estimated from the concentration gradient of N_2_O measured with a N_2_O microsensor (Andersen et al., 2001) in the tubes after acetylene treatment and establishment of anoxia (**Figure 2**). The rate of denitrification was calculated as the product of the surface area of the glass incubation chamber and the N_2_O flux, as described above for oxygen.

Three rounds of experiments were carried out using this setup. First, oxygen respiration rates were measured at 7°C in 6 batches of Greenland gromiids containing one, two or three cleaned specimens. These gromiids were also used in the second round of experiments, where denitrification rates on four batches of specimens, each containing 1–6 individuals were measured. Nitrous oxide production rates were measured during one to 8 h with intervals of 10 min and the rates given are average of the multiple measurements. Control experiments without acetylene were conducted to check for N_2_O production. After the microsensor measurements were terminated, the individual gromiids were removed from the incubation chamber and their dimensions measured.

A third round of experiments was carried out to test the sensitivity of denitrification to additions of antibiotics. Five batches of gromiids sampled in Uummannaq, Greenland (batch: V,X,Y,Z,) and one batch (batch L) sampled from Lillebælt, Denmark were used in these experiments. For each batch the following sequence of measurements was carried out: First, denitrification rates were determined and then a mix of the antibiotics ampicillin and streptomycin (which inhibit cell wall- and protein synthesis of many prokaryotes) was added to the incubation chamber, after which denitrification rate measurements were repeated. When these rate measurements were completed aerobic conditions in the microtubes were established by replacing the ascorbate in the cuvette with aerated seawater. Hereafter oxygen respiration rates were measured as described above. Finally the specimens were picked from the incubation chamber, their dimensions measured, and then they were frozen for later analysis of ICN.

Multiple measurements of denitrification rates were performed prior to the addition of antibiotics for periods of 2–20 h to ensure that inhibition of denitrification activity was not an artifact of long incubation periods. Denitrification activity was measured up to 20 h after the addition of antibiotics, and the rates given are averages of multiple measurements taken during measurement.

### Oxygen Concentration in Gromiids

Oxygen gradients within gromiids were measured directly at 7°C using an oxygen microsensor. Individual gromiids were placed in a glass capillary (0.55 mm i.d.) containing artificial seawater, and an oxygen electrode was slowly lowered into the interior of the cell. The spherical gromiids deformed and flattened at the basal side when pushed dorsally by the electrode and it was only possible to profile to the center of the specimen.

As an alternative approach, the intracellular oxygen concentration was calculated from the measured rates of oxygen consumption using a steady state-model describing the oxygen concentration in a sphere with zero-order kinetics for oxygen consumption:

(1) $$\begin{array}{c} C\left(r\right)=\frac{Q}{6D}\left(r^{2}-R^{2}\right)+C_{o}. \end{array}$$

Here, *C*(*r*) is the oxygen concentration at distance *r* from the center of a sphere, *Q* is the volume specific oxygen consumption rate, *R* is the radius of the sphere, and *C*_o_ is the oxygen concentration in the environment. *D* is the diffusion coefficient for oxygen at infinite dilution, corrected for salinity and temperature (Soetaert et al., 2016) and tortuosity. The tortuosity 𝜃 was estimated according to Boudreau (1997): 𝜃^2^= 1-ln(φ^2^), where φ is the porosity of the stercomata, which was assumed to be 0.7.

We further calculated critical radius which is the radius by which the oxygen concentration in the center of the sphere is zero. The critical radius *R*_crit_ can be determined from equation (1) as:

(2) $$\begin{array}{c} R_{\mathrm{crit}}=\sqrt{\frac{C_{o}\cdot 6D}{Q}}. \end{array}$$

## Results

### Intracellular Nitrate in Gromiids

All 107 individual gromiids tested in the present study contained nitrate at concentrations ranging from 0.7 to 80 mM, and the average ICN concentration for populations sampled in Arctic, temperate and Antarctic regions exceeded the ambient NO_3_^-^ concentration by a factor of 10^2^–10^4^ (**Table 1**).

**Table 1:** Intracellular nitrate content and concentrations in individual gromiids from different habitats, together with the bottom water NO_3_^-^ concentration.

Location	Individuals (#)	Intracellular nitrate content (pmol cell^-1^)			Volume (mm^3^)	ICN (mM)	Bottom-water NO_3_^-^ (μM)
		Mean (95% conf.)	Min	Max	Mean (SEM)	Mean (95% conf.)	
North Sea^a^	11	6259 (17343–2258)	158	47526	0.16 (3.5)	53 (19–153)	0–50^a^
Skagerrak Sweden^a^	12	6184 (26533–1441)	158	204000	0.51 (0.11)	16 (5–52)	30^a^
Skagerrak	3	853 (633–1150)	496	1255	0.61 (0.18)	1,5 (0.9–3)	11^b^
Kattegat	18	475 (920–245)	87	6928	0.67 (0.10)	1 (0.7–3)	11^b^
OMZ-Peru^a^	4	26458 (42150–16608)	13723	50000	0.1 (0.04)	370 (143–962)	15–40^a^
Bay of Biscay^a^	2	2846	1571	4121	0.093	35	20^a^
Rhône Delta^a^	13	2518 (4348–1458)	576	12676	0.16 (0.11)	46 (22–95)	5–10^a^
Disco bay Greenland^a^	13	9068 (14576–5641)	1205	35911	0.08 (0.02)	242 (105–561)	12^a^
Uummannaq Greenland	10	893 (2013–396)	50	500	0.06 (0.01)	24 (11–54)	<1^c^
McMurdo Sound Antarctica	21	134462 (257057–70334)	700	682880	4.47 (0.89)	49 (30–80)	30^d^

*The ICN content and concentration are reported as the geometric mean for the different habitats with the 95% confidence interval given in parenthesis. The volume of the gromiids investigated is reported as the arithmetic mean with the standard error of the mean given in parenthesis. ^a^Data from Piña-Ochoa et al. (2010), ^b^annual max data from Rysgaard et al. (2001) and Hansen (2015), ^c^measured at time of sampling, ^d^data from Barry (1988).*

### Sources of Intracellular Nitrate in Gromiids

The source of the ICN was investigated in a series of experiments where the ICN pool in individual gromiids was followed by adding ^15^NO_3_^-^ and ^15^NH_4_^+^ and by inhibiting nitrification with the addition of acetylene.

**FIGURE 3:**
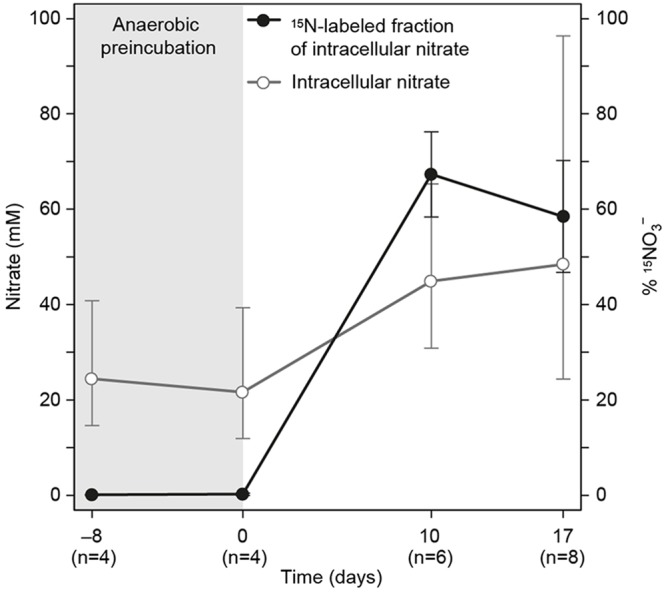
**Intracellular nitrate concentration and ^15^N labeling percentage in gromiids that were pre-incubated without nitrate and then incubated for uptill 17 days with 120 μM ^15^N labeled NO_3_^-^ in the media.** Error bars are 95% confidence intervals for ICN and standard error of the mean for labeling percentages. Numbers in paranthesis below the time axis refer to the number of individuals picked for analysis.

Active nitrate uptake was investigated in the ^15^NO_3_^-^ labeling experiment (**Figure 3**). The ^15^N atom % of the ICN pool increased significantly from background to 67 ± 9% (ave ± SEM) when gromiids were incubated with ^15^NO_3_^-^ and the positive correlation between ^15^N and ICN concentration and the incubation time was significant (Pearson; *r* = 0.81, *p* = 3.6 × 10^-5^, *n* = 17). The intracellular ^15^NO_3_^-^ concentration in specimens exposed to ^15^NO_3_^-^ for 7–14 days ranged between 4.5 and 113 mM which is more than 30-fold the concentration in the media (120 μM). The ^15^NO_3_^-^ uptake rate, estimated from the ^15^N enrichment of ICN of individuals, was highly variable, ranging from 25 to 3069 pmol N cell^-1^ d^-1^. The mean uptake rate was 703 ± 232 pmol N cell^-1^ d^-1^ (ave ± SEM). The ^15^NO_3_^-^ -uptake rates was significantly correlated with the cell volume of the individual specimens (Pearson; *r* = 0.80, *p* = 5.24 × 10^-4^, *n* = 14), suggesting that larger specimens had higher uptake rates than smaller ones. Normalized to cell volume, the ^15^NO_3_^-^ uptake rates ranged between 2.64 × 10^2^ and 6.63 × 10^3^ nmol cm^-3^ d^-1^. The mean was 2.53 × 10^3^± 5.5 × 10^2^ nmol mm^-3^ d^-1^ (ave ± SEM).

**FIGURE 4:**
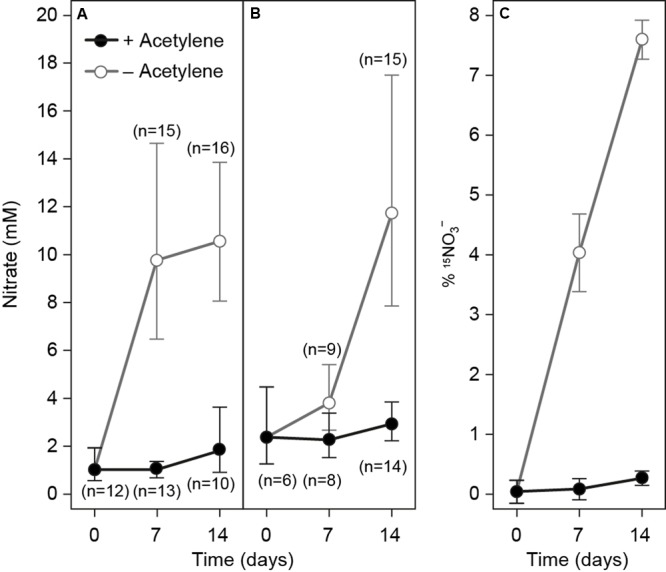
**Intracellular nitrate concentrations in gromiids that were incubated without nitrate in the media and either with (black circles) or without acetylene (Gray circles). (A)** No ammonia amendments. **(B)** Media supplemented with 50 μM ^15^NH_4_^+^ and **(C)** ^15^N labeling percentage of ICN in gromiids from the experiment shown in **(B)**. Error bars are 95% confidence intervals for ICN and standard error of the mean for labeling percentages. Numbers in parenthesis refer to the number of individuals picked for analysis.

The production of nitrate in gromiids was studied by following the ICN concentrations over time using specimens incubated in nitrate-free media with and without acetylene (**Figure 4**). Gromiids incubated without acetylene and without NH_4_^+^ for 1–2 weeks had significantly higher ICN concentrations than those incubated in media with acetylene and without NH_4_^+^ (*t*-test; *p* = 4.34 × 10^-13^ *n* = 54). For gromiids incubated without acetylene the ICN concentration was significantly and positively correlated with incubation time (Pearson *r* = 0.68, *p* = 4.19 × 10^-7^; *n* = 43), whereas no significant relationship between incubation time and the ICN concentration were seen for specimens incubated with acetylene (Pearson; *r* = -0.017, *p* = 0.93, *n* = 35). Similar results were obtained when analyzing the ICN content – treatment relationships.

Incubations performed with ^15^NH_4_^+^ demonstrated a capacity for IC^15^N production only in specimens incubated without acetylene (**Figure 4B**). As above, the ICN concentration was significantly higher in specimens incubated without acetylene than in specimens incubated with acetylene (*t*-test; *p*-value = 1.68 × 10^-5^, *n* = 46), and a significant correlation between incubation time and ICN concentration was seen only for specimens incubated without acetylene (Pearson; *r* = 0.68, *p* = 3.0 × 10^-5^, *n* = 30). The intracellular concentration of ^15^NO_3_^-^ was likewise significantly higher in the specimens incubated without acetylene, than in specimens incubated with acetylene (*t*-test; *p* = 1.96 × 10^-2^, *n* = 46) and there was a significant positive correlation between incubation time and IC^15^N concentration for specimens incubated without acetylene (Pearson; *r* = 0.92; *p* = 9.3 × 10^-13^, *n* = 30); no such correlation was seen for specimens incubated in the presence of acetylene (Pearson; *r* = 0.33; *p* = 0.082, *n* = 28). The mean rate of combined uptake and nitrification of ^15^NH_4_^+,^, calculated from the ^15^N enrichment of the ICN pool in individuals was 46 ± 8 pmol gromiid^-1^ d^-1^ for specimens incubated in the absence acetylene (this rate should be considered as a minimum estimate of nitrification as it does not include the nitrification of ^14^NH_4_^+^ eventually present in the cells). There was significant correlation between cell volume and the rate of nitrification of ^15^NH_4_^+^ (Pearson, *r* = 0.50; *p* = 0.014, *n* = 24). The mean volume specific rate was 61 nmol cm^-3^ d^-1^± 14 nmol cm^-3^ d^-1^ For specimens incubated in the presence of acetylene the mean rate calculated with this procedure was 0.12 ± 0.11 pmol gromiid^-1^ d^-1^, which is not statistically different from zero (*t*-test; *p* = 0.295, *n* = 22). For all samples nitrate concentrations in the media were below 2 μM and ^15^NO_3_^-^ was below the detection limit.

### Aerobic Respiration and Intracellular Oxygen Concentration in Gromiids

Oxygen microprofiles above gromiids placed in a glass capillary showed clear evidence for oxygen consumption (Supplementary Figure S1). The rate of oxygen consumption ranged from 574 to 2127 pmol O_2_ cell^-1^ d^-1^ (Supplementary Table S1) and was significantly correlated with the cell volume of the specimens measured (Pearson, *r* = 0.96; *p* = 2.18 × 10^-3^, *n* = 6). The average volume specific oxygen consumption rate was 3.29 × 10^5^± 3.05 × 10^4^nmol cm^-3^ d^-1^.

**FIGURE 5:**
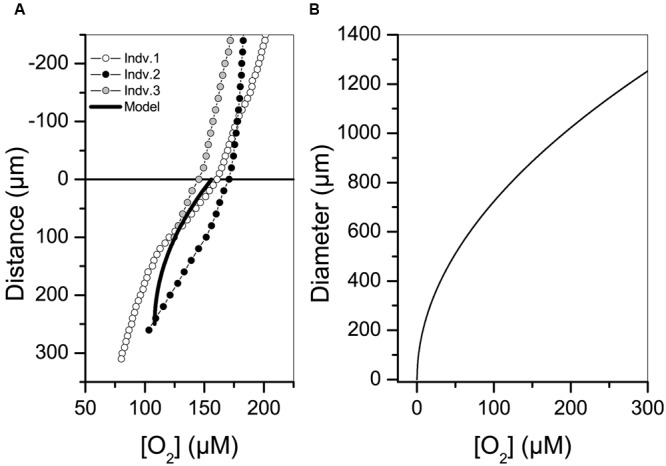
**(A)** Intracellular oxygen concentration profiles in three gromiid specimens each having a diameter of approximately 500 μm. Zero marks the surface of the gromiid and positive numbers show the distance from the surface into the gromiid cell body. The modeled oxygen concentration is shown as a black line. **(B)** Diameter at which the oxygen concentration reaches zero in the center of a spherical cell in relation to ambient oxygen concentration.

Oxygen concentration within the gromiid cell body results from oxygen consumption by cellular respiration and transport of oxygen to and within the cell by diffusion. The intracellular oxygen concentration was measured by oxygen microelectrodes and was also modeled using the average volume specific oxygen consumption rate above (**Figure 5**). Profiling on three separate gromiids having a diameter of approximately 500 μm showed that these specimens were oxic throughout the interior. Results from the modeled intracellular oxygen concentrations fairly reproduced these profiles and indicated that only large gromiids with a diameter above 1000 μm can turn anoxic centrally at full oxygen saturation of the ambient waters.

### Denitrification of Intracellular Nitrate

All four batches of gromiids which were tested for denitrification of the ICN produced nitrous oxide under anoxic conditions (Supplementary Figure S2). The fact that nitrous oxide was not detected without acetylene suggested that the nitrate was completely denitrified to N_2_. Denitrification rates calculated from the nitrous oxide profiles ranged from 19 to 171 pmol N cell^-1^ d^-1^ (Supplementary Table S2) and there was no clear relationship between cell volume and denitrification activity; including data from the subsequent antibiotic experiments (**Table 2**) did not improve this correlation (Pearson *r* = 0.057, *p* = 0.8829, *n* = 9).

**Table 2:** Denitrification rates measured before and after addition of antibiotics together with aerobic respiration rates and intracellular nitrate concentration.

Batch	Individuals #	Total biovol. (mm^-3^)	Denitrification (pmol N ind^-1^d^-1^)	Denitrification w. Antibiotic (pmol N ind^-1^d^-1^)	O_2_ respiration (pmol O_2_ ind^-1^d^-1^)	O_2_ respiration (nmol cm^-3^ d^-1^)	ICN (mM)
V	3	3.30 × 10^-2^	89.9 (2.19)	-2.45 (2.55)	1352 (19.4)	1.23 × 10^5^ (8.82 × 10^2^)	36 (16–81)
X	4	4.00 × 10^-2^	9.75 (3.28)	0.94 (1.32)	1359 (18)	1.36 × 10^5^ (9.00 × 10^2^)	34 (10–118)
Y	4	5.81 × 10^-2^	2.71 (1.179)	0.042 (0.479)	N.D.	N.D.	3.44 (0.18–64.36)
Z	4	1.41 × 10^-1^	17.0 (0.923)	1.11 (0.62)	N.D.	N.D.	11.46 (6.28–20.94)
L	3	7.08 × 10^-2^	695 (172)	3.86 (7.12)	1259 (100.16)	5.33 × 10^4^ (2.12 × 10^3^)	1.19 (0.59–2.89)

*The ICN is reported as the geometric mean with the 95% confidence interval given in parenthesis. The total biovolume is the sum volume of all cells in a given batch.*

The effect of antibiotics on denitrification rates was tested on five batches of gromiids in a separate round of experiments. During these experiments denitrification was monitored by repeated profiling over periods of 2–20 h prior to addition of antibiotics. All five batches produced N_2_O during this period, though with highly variable rates (**Table 2** and Supplementary Figure S3). The addition of antibiotics inhibited N_2_O production within 24 h in all batches investigated. The response times and response patterns, however, were highly variable. In the batch with lowest denitrification activity (Y), N_2_O production was below detection limit 5 h after antibiotic addition, whereas for the V and the L batches, N_2_O production continued at highly variable intensity for up to 20 h in the presence of antibiotics (Supplementary Figure S4). When conditions in the incubation chambers turned oxic after antibiotic treatment, oxygen profiling showed that aerobic respiration was reestablished (Supplementary Figure S5). The aerobic respiration rates measured in the presence of antibiotics (**Table 2**) were significantly lower than respiration rates reported above, measured on untreated individuals (*t*-test; *p* = 0.0010). All gromiids used in these experiments contained nitrate at concentrations >1 mM at the end of the experiments (**Table 2**).

## Discussion

### Intracellular Nitrate in Gromiids

A review of the nitrate content in gromiids tested for ICN (**Table 1**), shows that the capacity to store nitrate intracellularly, at levels far above the ambient nitrate concentration, is a ubiquitous and an obligate trend within *Gromia* and not just a property of specimens from a particular habitat. Gromiids from Artic, Antarctic, temperate and tropical regions all had high ICN pools, and within each of these geographically distributed populations we never encountered individuals without ICN. The high ICN concentrations in gromiids are comparable to those found in some foraminifera (Piña-Ochoa et al., 2010) and diatom species (Dortch et al., 1984; Lomstein et al., 1990; Kamp et al., 2011, 2013). By contrast, however, ICN storage within those taxa is not obligate, since species without ICN are recurrently encountered (Dortch et al., 1984; Piña-Ochoa et al., 2010; Langlet et al., 2014).

### Sources of ICN in Gromiids

The results of our experiments suggest that the presence of ICN in gromiids can result from two different mechanisms: (1) ICN production and (2) active nitrate uptake. The capacity for ICN production was evidenced from experiments performed with gromiids incubated in the absence nitrate in the environment. Here ICN was produced, but only in the absence of acetylene (**Figure 4**). This ICN production presumably takes place through bacterial-mediated nitrification since (a) this process is inhibited by acetylene, which is known to block ammonium monooxygenase (Hyman and Wood, 1985), and (b) since labeled nitrogen added as ammonium could be found in the ICN pool (**Figure 4B**). Further analysis of the microbial community is, however, required to confirm the presence of active nitrifying bacteria within gromiids.

The presence of an active NO_3_^-^ uptake mechanism by the gromiid cell was evidenced from the results of ^15^NO_3_^-^ labeling experiments (**Figure 3**). Here, all individual specimens investigated took up ^15^NO_3_^-^ to concentrations that by far exceeded the ambient concentration of ^15^NO_3_^-^. This result excludes passive uptake of nitrate from the media via diffusion across the cell membrane. Such evidence for an active uptake of nitrate has also been found in other nitrate-storing protists like diatoms and foraminifers (Risgaard-Petersen et al., 2006; Kamp et al., 2013).

### Sinks for ICN in Gromiids

From N_2_O accumulation experiments performed in the absence or presence of acetylene we identified a complete denitrification pathway within gromiids in which ICN is reduced to N_2_. A capacity for complete denitrification has also been found in various benthic foraminifers within the order Rotaliida (e.g., Risgaard-Petersen et al., 2006; Høgslund et al., 2008; Piña-Ochoa et al., 2010; Bernhard et al., 2012a), and the rates measured in the gromiids here fall within the range reported for these rotalids (i.e., 40–1992 pmol cell^-1^ d^-1^; Kamp et al., 2015). However, while the variation in denitrification rates among foraminifers can be explained by variations in cell size (Kamp et al., 2015), such a correlation was not observed for the gromiids investigated in the present study. This finding could suggest that denitrification is not indigenous to the metabolism of gromiids, but is rather a consequence of harboring a denitrifying microbial community with a more random size and activity in the sedimental and detrital material, that it encapsulates. Indeed, results of the antibiotic experiments performed in the present study (**Table 2**) suggest that denitrification in gromiids, like in the Santa Barbara Basin allogromiid (Bernhard et al., 2012b), was performed by associated bacteria and not by the eukaryotes themselves. As indicated in **Table 2** and Supplementary Figures S3, S4, the application of ampicillin and streptomycin to gromiids led to halt of denitrification which is in contrast to observations made for a denitrifying rotaliid foraminifer, where bacteria specific antibiotics had no effect on the ability of the organism to denitrify (Bernhard et al., 2012a). That the antibiotics applied in the present study were specific for bacteria present within the gromiid was indicated by the observation that the gromiids could respire oxygen and retain the ICN-pool following antibiotic treatment.

### Role of ICN in Gromiids

The role that ICN plays in gromiid autecology is enigmatic. First, gromiids are generally found in oxic environments like sediment surfaces or on seaweed hapterans in the intertidal (Arnold, 1972). This habitat preference may exclude the need for alternative electron acceptors like nitrate in respiration. Furthermore, although a capacity for nitrate respiration via denitrification is present within gromiids, our results indicate that the energy delivered from this process bypasses the gromiid because denitrification, if expressed, is catalyzed by bacteria. It is possible that gromiids make use of their ICN as a nitrogen source, as seen for example in brown algae (Young et al., 2007). It is also possible that the ICN may serve as a sulfide buffer in case gromiids face anoxia. From a thermodynamic point of view, denitrification is more favorable than sulfate reduction and by maintenance of ICN gromiids may thus reduce the possibility of intracellular sulfate reduction by bacterial endobionts, hence preventing sulfide accumulation within the cell. A third possibility is that ICN-based denitrification could act as a sink of fermentation products produced by the gromiid and/or the endobiotic bacterial community in case they encounter anoxic conditions.

Gromiids may face anoxia either if the surrounding environment turns hypoxic (e.g., if the specimen is buried by sediment; Gooday et al., 2013) or if the large gromiid cell inflicts anoxia on itself. Self-inflicted anoxia may arise if the oxygen demand of the cell exceeds the diffusional supply of oxygen and thus depends on the activity and diameter of the cell (**Figure 5B**). We note that the cell-interior was oxic in specimens with a diameter of 500 μm when ambient oxygen concentrations were 180 μM (**Figure 5A**), but we cannot exclude the possibility of intracellular anoxia from cellular respiration. The gromiid will turn anoxic if surrounding oxygen concentrations decreases or if cell diameters approach 1 mm, when respiration activities are within the range measured in the present study (**Figure 5B**). The nitrogen cycle mediated by gromiids and their endobiotic community appears, therefore, not only to be dependent on the oxygenation status of the surrounding environment, but also on the respiratory activity of the cell and its accompanying endobionts. Alternations between anoxic and oxic conditions in the gromiid would promote an intercellular nitrogen cycle of combined nitrification and denitrification mediated by endobiotic bacteria. Whether or not a gromiid actively takes part in regulating the intracellular bacterial community or passively carries bacteria taken up from the environment is an intriguing question that requires further study.

## Author Contributions

SH and NR-P designed the research and interpreted results. SH performed the experiments. SH, TC, and SB performed the *in situ* sampling of gromiid specimens. SH and NR-P wrote the paper with significant intellectual and linguistic input from SB and TC.

## Conflict of Interest Statement

The authors declare that the research was conducted in the absence of any commercial or financial relationships that could be construed as a potential conflict of interest.
