# Supplementary material for: Sinks and Sources of Intracellular Nitrate in Gromiids
Source: Front Microbiol. 2017 Apr 20;8:617. doi: 10.3389/fmicb.2017.00617 (PMC5397464; doi:10.3389/fmicb.2017.00617)
Supplement: Supplementary file 1 [file Data_Sheet_1.docx]

**Sinks and Sources of Intracellular Nitrate in Gromiids**

**Supplementary Information**

Signe Høgslund^1^, Tomas Cedhagen^1^, Samuel S. Bowser^3^ & Nils Risgaard-Petersen^1,2*^

^1^Department of Bioscience, Aarhus University, Aarhus, Denmark

^2^Center for Geomicrobiology, Aarhus University, Aarhus, Denmark

^3^Wadsworth Center, New York State Department of Health, Albany, NY, USA

**Figure S1:** Oxygen concentration profiles in micro-tubes loaded with gromiids from batch D-I. Data points represent the average of 4 measurements, with the exception of the profile for batch I, where 10 profiles were made. Error bars represent the standard error of the mean.

**Table S1:** Oxygen consumption rates of gromiids from batch D-I as estimated from the oxygen profiling. Standard errors of the mean are given in parenthesis. The total bio volume is the sum volume of cells in given batch.

| Batch | #individuals | Total biovolume  (mm^-3^) | Cell specific oxygen consumption  (pmol O_2_ cell d^-1^) | Volume specific oxygen consumption  (nmol O_2_ cm^-3^ d^-1^) |
| --- | --- | --- | --- | --- |
| D | 3 | 7.69 x 10^-3^ | 791 (12) | 3.09x10^5^ (4.54x10^3^) |
| E | 2 | 4.97 x 10^-3^ | 575 (3) | 2.31x10^5^ (1.02x10^3^) |
| F | 1 | 3.22 x 10^-3^ | 1143 (42) | 3.56x10^5^ (1.31x10^4^) |
| G | 3 | 3.91 x10^-3^ | 595 (51) | 3.04x10^5^ (2.60x10^4^) |
| H | 2 | 4.30 x 10^-3^ | 984 (15) | 4.57x10^5^ (7.12x10^3^) |
| I | 1 | 1.34 x 10^-2^ | 2128 (61) | 3.18x10^5^ (9.12x10^3^) |
|  |  |  |  |  |


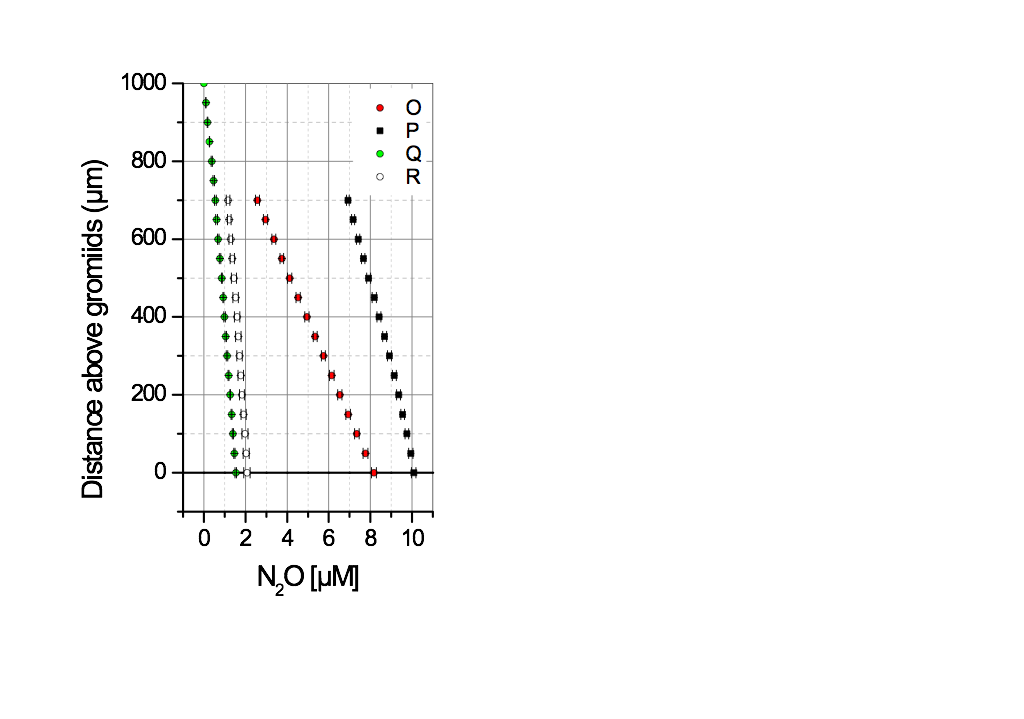


**Figure S2:** Nitrous oxide concentration profiles in micro-tubes loaded with gromiids from batch O-R. Each data point represents the average of 4 measurements. Error bars are the standard error of the mean.

**Table S2:** Denitrification rates of gromiids from batch O-R as estimated from the N_2_O-profiling. Standard errors of the mean are given in parenthesis. The total bio volume is the sum volume of cells in a given batch.

| Batch | #individuals | Total bio volume  (mm^-3^) | Cell specific denitrification rate  (pmol N cell d^-1^) | Volume specific denitrification rate  (nmol N cm^-3^ d^-1^) |
| --- | --- | --- | --- | --- |
|  |  |  |  |  |
| O | 4 | 5.20 x 10^-2^ | 171 (1.0) | 1.32x10^4^(7.31 x 10^1^) |
| P | 3 | 2.90 x 10^-2^ | 144 (7.0) | 1.49x10^4^ (7.24 x 10^2^) |
| Q | 1 | 1.80 x 10^-2^ | 84 (9.5) | 4.67x10^3^ (5.28 x 10^2^) |
| R | 6 | 6.80 x 10^-2^ | 19 (1.8) | 1.68x10^3^(1.59 x 10^2^) |
|  |  |  |  |  |


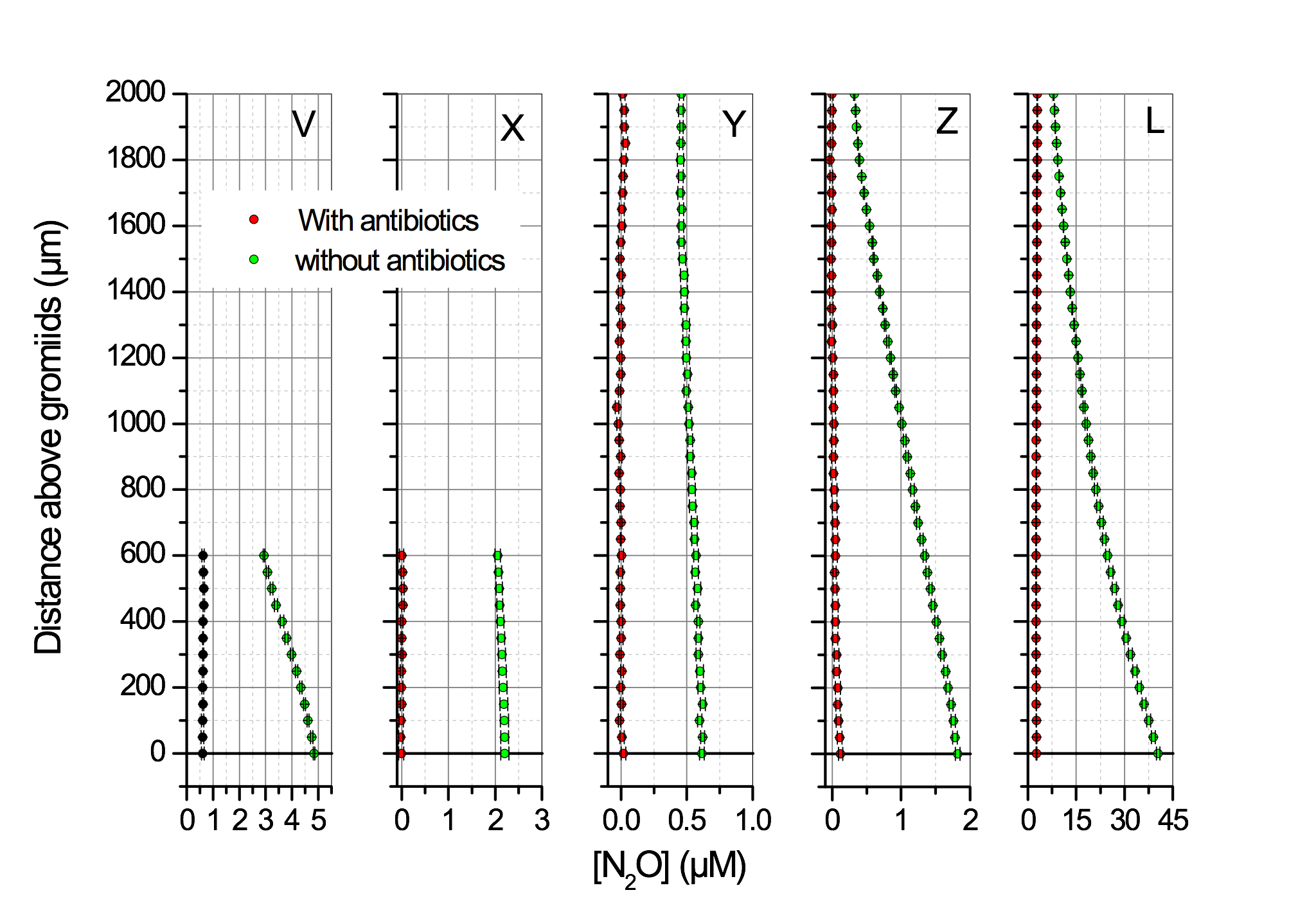


**Figure S3:** Nitrous oxide concentration profiles in micro-tubes loaded with gromiids from batch V-L. The profiles shown were measured approximately 1 hour prior to antibiotic treatment (green dots) and 5 h (batch Y), 19 h (batch L), 20 h (batch V), and 24 hours (batch Z and X) after (red dots). Each data point represents the average of 4 measurements. Error bars are the standard error of the mean.


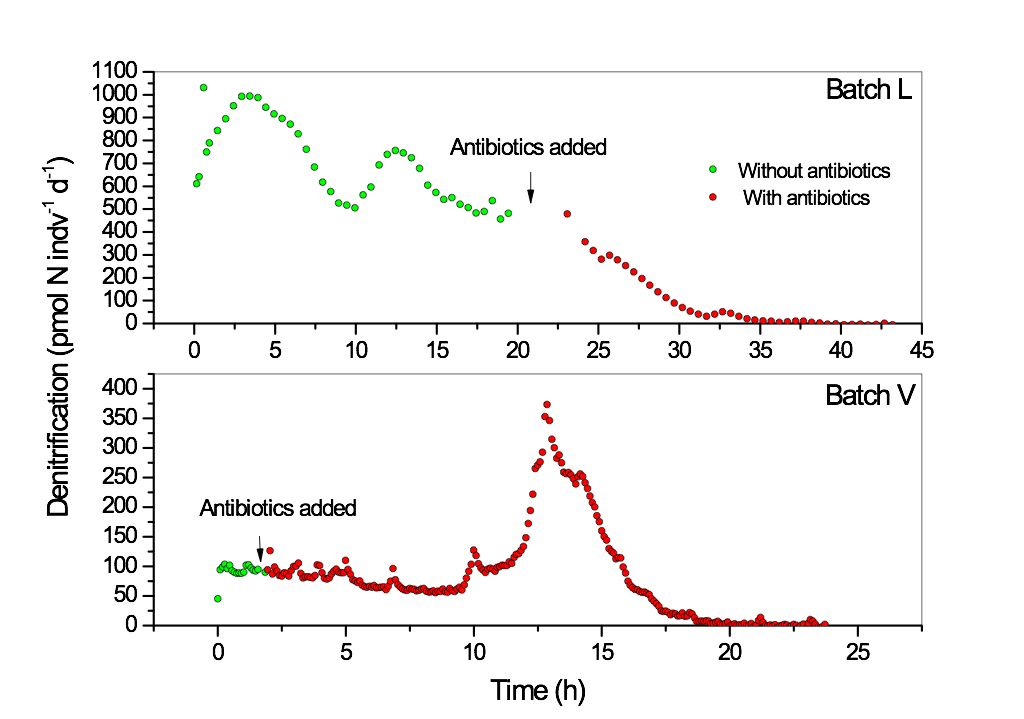


**Figure S4:** Denitrification rates estimated from N_2_O concentration profiles measured in microtubes loaded with gromiids from batch L and V. Green dots represent rates measured prior to the addition of antibiotics (ampicillin and streptomycin); red dots show after the addition.


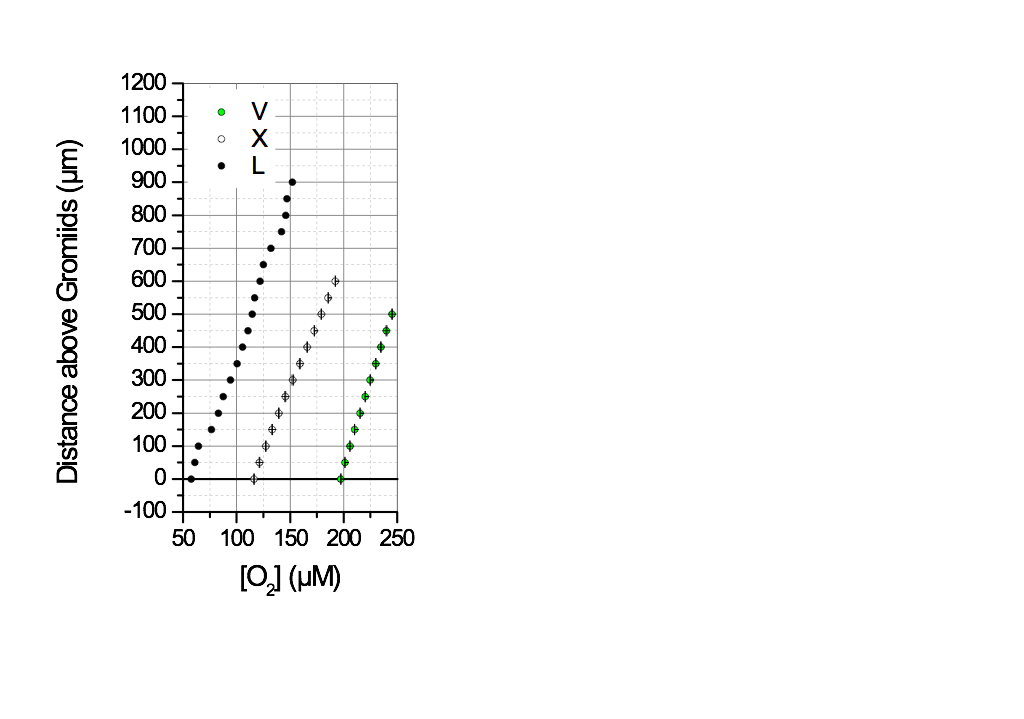


**Figure S5**: Oxygen concentration profiles in microtubes loaded with gromiids from batch V, X, L. Profiles were measured after the conditions in the incubation chambers turned oxic after antibiotic treatments. Data points represent the average of 3 measurements. Error bars represent the Standard Error of the mean.
